# Miscellaneous Topics

**Published:** 1906-04

**Authors:** R. F. Sheehan

**Affiliations:** Buffalo


### PROGRESS IN MEDICAL SCIENCE.

### Miscellaneous Topics

By R. F. SHEEHAN, Buffalo.

### INDOOR HUMIDITY

The humidity of the air within doors, (Bulletin Chicago Health Department, Nov. 18, 1905) particularly when heated by hot air furnaces, averages at this time of the year, when the windows are closed for the major portion of the day, less than 30 per cent., while the average humidity of the atmosphere, though it may vary within wide limits, is approximately 60 per cent. even upon the brightest and most sunshiny days. It is safe to say that this sudden and violent contrast is almost certain in many people to work harm particularly upon the delicate mucous membrane of the upper respiratory tract.

Dr. H. M. Smith, who read a paper on this subject before the Brooklyn Medical Society, 1904, believes that the neglect of providing the element of watery vapor in the air as the greatest cause of our over heating our homes, and further that a low humidity is the great cause of discomfort and the source of much ill health, as in the production of coughs and colds. Many experiments have shown that a humidity above 50. with a temperature about 65.°,yields the maximum amount of comfort, and are the conditions best compatible with health. On the other hand, a humidity of 30 with a temperature of 75°as compared with the former affords a less degree of pleasurable comfort.

Wilson says "about 25 per cent. of the cost of heating is expended in raising the temperature from 60° to 70°, so if we can keep comfortable at a temperature of 65° we shall save 12½ per cent. of the total cost of heating." In other words we are wasting fuel to an enormous extent in maintaining an unnecessarily high temperature. Smith further adds: "It is most interesting and instructive in connection with this matter to find that on the perfect days in May and early June, with all the windows open admitting freely the outside air, the thermometer stood at 65° to 68° and the hygrometer registered about 60 per cent. relative humidity."

A moments thought recalls the fact that we often sit out of doors with perfect comfort at a temperature that would cause us to shiver in our rooms in winter. The relative humidity is the balance-wheel that regulates our comfort at different temperatures in the still air. The lesson to be drawn from these facts is that if a room heated to from 65° to 68° is too cold for a healthy person, the relative humidity and not the temperature should be raised.

### SPRAINED ANKLE

Robert Carothers (*Lancet Clinic,* Dec. 2, 1905) makes the following conclusions on this subject:

I. That no one is exempt from a sprained ankle, although some are more prone than others.

II. That in severity a sprained ankle will range from a trivial accident to one of extreme severity and everlasting.

III. That the outer side more often than the inner side of the ankle is the seat of the trouble.

IV. That the diagnosis, which is ordinarily made with ease, is at times made with difficulty, and at times again, an *x*-ray examination is required to make the diagnosis certain.

V. That the treatment by immobilization with a plaster-of-paris cast is unsatisfactory and at times injurious.

VI. That the treatment instituted by Cotteral, the so-called adhesive plaster strappings in the manner described, advising and urging the patient to walk on the injured foot, the early removal of these straps followed by massage, gives the most satisfactory and best results.

VII. That the old cases are to be, under anesthesia, converted into acute sprains, and treated in the same manner.

### THE TOXIC AGENT OF LOBAR PNEUMONIA-THERAPEUTICS

Synopsis of an exhaustive paper by Walter V. Brem, Jr.,M.D. (Bulletin of the Johns Hopkins Hospital, October, 1905).

I. *Action of the toxic agent of lobar pneumonia.*

a. Phenomena of the mild action bear the features of stimulation of the central nervous system and cardiac muscles.

b. Phenomena of the severe intoxication appear to result from intensified stimulation, or enfeeblement and exhaustion from overstimulation.

c. Death occurs from (1) respiratory insufficiency terminating in asphyxiation, or in exhaustion of the respiratory center, or (2) circulatory insufficiency which leads, presumably, to accumulation of the toxic agent, and which may induce edema of the lungs, or end in exhaustion of the heart muscle.

II. *Therapeutics.*

First, elimination of the toxic agent. Internal hydrotherapy.

Second, amelioration of harmful influences.

a. Fever—external hydrotherapy. Pain—ice bag and analgesics. Restlessness, insomnia, delirium—external hydrotherapy, analgesics and narcotics.

b. Respiratory indications.

1. Heroin or morphine every two hours for a respiratory rate of thirty-six or greater.

2. Oxygen inhalation is probably useless and may be harmful.

c. Circulatory indications:

1. Circulatory sedatives probably contraindicated, excepting the nitrates, which may be of benefit during the early periods of increased cardiac work.

2. Alcohol indicated in alcoholic cases, may be of benefit when there is no circulatory insufficiency.

3. Circulatory stimulants contraindicated, except members of the digitalis series. The indication is low blood pressure associated with one or more of three conditions—namely, respiratory insufficiency, small urinary output and edema of lungs.

---

VITALITY OF TUBERCLE BACILLI IN SPUTUM

D. C. Twitchell (*Medical News,* Sept. 30, 1905) summarises his experiments as follows:

I. Darkness and moisture are most conducive to the prolonged life of the tubercle bacilli in sputum, under these conditions remaining alive at the end of five months and a half.

2. Dryness hastens their destruction. A temperature of 37° is less favorable for them than ordinary room temperature. A temperature near the freezing point is less favorable for them than ordinary room temperature.

3 The direct rays of the sun kill them in a few hours.

